# Using learning profiles to inform education priorities: An editors’ overview of the Special Issue

**DOI:** 10.1016/j.ijedudev.2021.102477

**Published:** 2021-10

**Authors:** Luis Crouch, Michelle Kaffenberger, Laura Savage

**Affiliations:** a RTI International, United States; b University of Oxford, United Kingdom; c Foreign, Commonwealth, and Development Office, Government of the United Kingdom, United Kingdom

**Keywords:** Learning profiles, Learning crisis, Education systems, Foundational skills, Inequality

## Abstract

This special issue explores the use of learning profiles for analysing the dynamics of low learning in low- and middle-income countries and informing priorities to address the learning crisis. The 12 papers in the special issue draw on learning data from more than 50 countries and 6 million individuals, with implications for education policy and practice. Taken together, they point to a need to steepen learning trajectories by prioritizing early mastery of foundational skills for all children. The papers show that addressing the learning crisis will not be achieved through more school grade attainment alone, nor through within-country equality across groups (such as girls and boys or rich and poor). Positive examples show that programs focused on foundational learning both improved average learning and reduced inequality. Addressing the learning crisis will require a focus on systems improvement, using foundational learning as a case in point for making the needed systems improvements to steepen learning throughout children’s time in school. Learning profiles can provide a guide for education actors aiming to improve learning outcomes.

## 1 Introduction

*“Shall I tell you about a granny I know? She’s a really strange old lady and so full of life!”*^1^ We know that only about half of 10-year-old children in low- and middle-income countries can read and understand sentences like these two (World Bank, 2019). When in their schooling did things begin to go wrong, leaving so many unable to read? Are there any promising signs of learning improvements over time? Who, among these cohorts of children, is progressing? And how can we better understand why learning levels for most are so low, and persistently so?

This Special Issue presents 12 papers taking various approaches to, and highlighting different uses of, *learning profiles*. Altogether they draw on learning data from more than 50 countries and 6 million individuals to examine the breadth and depth of the learning crisis and inform priorities to address it.

Better understanding of the dynamics of low learning are needed both at the national and international level. Despite growing acknowledgement of poor student learning outcomes in low- and middle-income countries (LMICs), many efforts and much dialogue still focus narrowly on expanding access to schooling. While statistics on low learning periodically make headlines, the depth and the scale of the crisis are still not well understood.

Learning profiles help meet this need. Learning profiles represent the dynamics of children’s learning, showing the connection between ages or grades and the achievement of any skill, capability, or competency (Kaffenberger, 2019). While most learning assessments cover only in-school children of a single age or grade, learning profiles use data across multiple ages or grades, giving insight on children’s learning *progression* as they advance through school. Because learning profiles typically use data that covers a full cohort of children (or adults), including those who dropped out of school, learning profiles also give insight on progress towards *universal* learning goals, and what it will take to reach universal goals.

Through analysis of learning profiles, education leaders and advisors can understand the dynamics of learning and learning inequalities in their country, identify groups of children who are behind and when in their schooling career they began to fall behind, and observe education systems’ progress towards meeting their own education goals. Analysis of learning profiles can also help identify positive and negative deviance examples - where learning gains are particularly steep or flat.

The papers in this Special Issue use learning profiles to help us understand how much – and how little – students are learning over the course of their schooling career in many LMICs. They use learning profiles to examine the role of increasing schooling grade attainment (versus steepening learning) to address the learning crisis; the sources of inequalities in learning outcomes and how to improve learning equity; and the effects of foundational-learning-focused programs on inequality. The papers also model different policy approaches to improving learning; assess the reliability of different sources of learning data; and analyze some of the few panel data learning assessments available in LMICs.

The data needed to build learning profiles has been limited in LMICs until recently. The papers in this Special Issue capitalize on the growing availability of such learning data, often drawing on non-traditional sources of learning assessments. The papers and their findings collectively have five key implications for education policy and practice.

## 2 Five implications from the Special Issue papers

### 2.1 The learning crisis is worse than we think it is, and more years of schooling will not resolve it

**Fig. 1:**
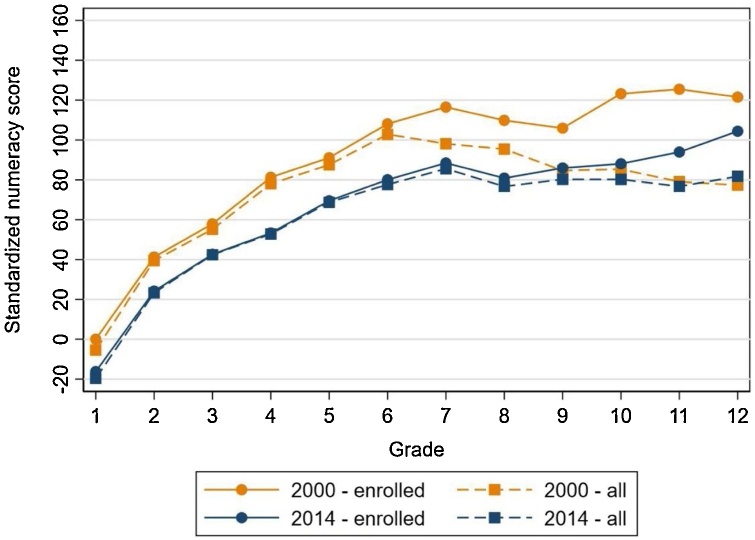
Learning profiles for foundational numeracy skills in Indonesia flattened after grade 6, and learning profiles were lower in 2014 than in 2000. *Note:* Standardized numeracy score (rescaled to have a mean of 0 and standard deviation of 100 for grade 1 students in 2000; scores are in terms of grade 1 (in 2000) standard deviations) in 2000 and 2014 by grade level completed (for enrolled children) or grade level they would have completed (for all enrolled and unenrolled children), using the Indonesia Family Life Surveys (IFLS) data. Between grades 6 and 12 enrolled students gain only 0.2 standard deviations.

*Source*: Beatty et al. (this issue).

The analysis in this Special Issue shows that the majority of children in LMICs are learning very little as they progress from one year to the next, leaving them without the most basic foundations of reading and arithmetic even after spending many years in school.^2^ Among young women who completed primary school (and no higher) across 50 countries, only half could read a single, simple sentence despite their many years spent in school (Pritchett and Sandefur, this issue). Similarly, across 10 LMICs, only half of young men and women who had completed primary school could read a simple passage in a household-based reading assessment (Kaffenberger and Pritchett, this issue, b). Learning profiles also often flatten as children progress through school, indicating that children who do not gain foundational skills in the early grades frequently do not gain them later (Fig. 1) (Beatty et al., this issue; Muralidharan and Singh, 2021).

In some places learning profiles have been getting worse, not better, in recent years (Beatty et al., this issue; LeNestour et al., 2021). Beatty et al. (this issue) find that between 2000 and 2014 mathematics test scores in Indonesia declined by one-fourth of a standard deviation (Fig. 1). The average child in grade 7 in 2014 had learned as much as the average child in grade 4 in 2000, and all subgroups were affected by the declines.

Furthermore, it is likely that even the low estimated learning per year in many places is often biased upwards. A study of Rwanda found that once selection effects were addressed,^3^ learning per year was even lower than previously thought (Crawfurd, this issue). Johnson and Parrado (this issue) find that learning outcomes in India are likely much lower than national assessments suggest and, just as importantly, suggest that learning outcomes are hard to understand because the official data seem quite unreliable, especially at the lower end of performance.

**Fig. 2:**
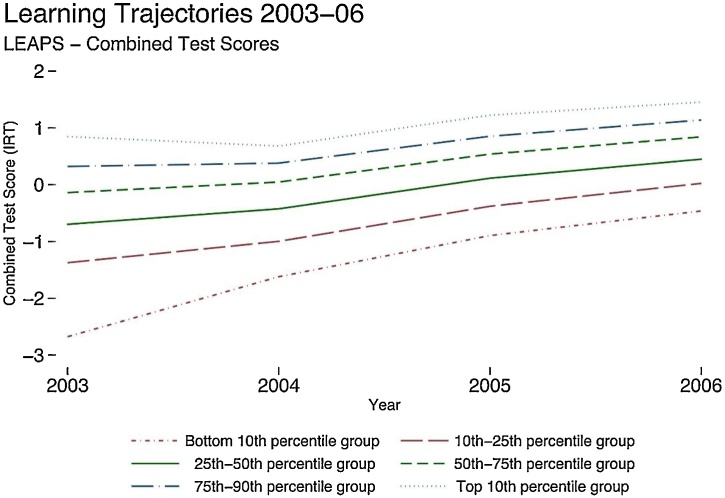
Learning profiles in Punjab, Pakistan, reveal convergence between initially low and high performers in later primary school. *Note:* This figure shows learning trajectories by groups of baseline levels of test score performance during Grade 3–6 using the unbalanced full sample but restricting the graph for those who were observed in Grade 3 (2003). The graph shows averaged test scores across the three subjects tested for children at different test scores levels in 2003.

Some of the studies show more promising trends. In Pakistan, learning profiles show low performers converging with high performers in the late primary years (Fig. 2) (Bau et al., this issue).^4^

*Source:*Bau et al. (this issue).

Because of relatively flat learning profiles, in many countries little learning would be gained even through many more years of schooling, according to simulations of learning under universal school completion (Kaffenberger and Pritchett, this issue, b; Kaffenberger and Pritchett, this issue, a; Pritchett and Sandefur, this issue). One paper suggests a 70-percentage-point increase in secondary school completion (to universal) in LMICs would have no impact on the percent of children reaching Sustainable Development Goal 4 of minimum proficiency in mathematics (Kaffenberger and Pritchett, this issue, a): learning profiles are too flat for children to reach this goal even with many additional years in school.^5^

Context also matters. The simulated effects of expanding schooling vary widely – expanding schooling to universal primary completion in Ethiopia would improve women’s literacy by 57 percentage points, to 75 percent literate, and thus prioritizing more schooling could yield substantial gains (Pritchett and Sandefur, this issue). In Nigeria, however, literacy is projected to increase by only 5 percentage points under universal primary schooling, so prioritizing steepening learning profiles would yield much more learning. Different education systems clearly need different approaches and learning profiles can help inform priorities.

### 2.2 Achieving equitable outcomes requires raising the floor on learning levels, starting with foundations

**Fig. 3:**
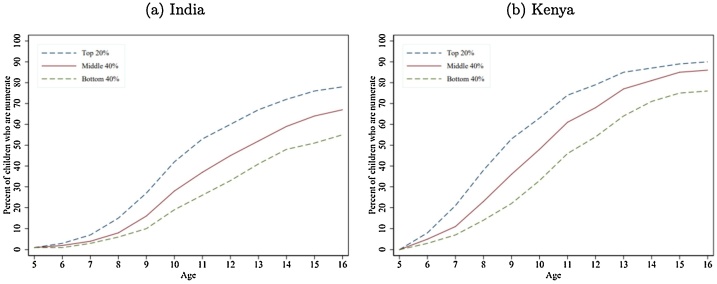
At age 10, even among the top 20 % by SES, less than 60 % of children have achieved basic numeracy in India and Kenya. *Note:* Learning profiles show percent of children at each age demonstrating grade 2 numeracy proficiency, based on ASER and Uwezo data.

*Source*: Akmal & Pritchett (this issue).

In many countries, learning is low for nearly all children. Therefore, focusing on targets for equality across groups, such as rich and poor, *within* countries, will not do enough to improve equality of learning *between* countries or to help poorer countries catch up to a global target. Akmal and Pritchett (this issue) find that, across five LMICs, even if the poorest children were supported to learn as well as the richest, universal mastery of basic skills such as literacy would still be a distant goal (Fig. 3).

Other papers find that within countries, the largest source of inequality is between bottom and top performers, not across commonly identified characteristics such as gender, income, or geography (Crouch et al., this issue). Combining these two points suggests that an explicit focus on mastery (and starting with the foundations), full stop, is needed, as well as a focus on low performers as such, rather than only by income group, gender, etc.

Several papers point to efforts that prioritize foundational skills for all children as a key way to both improve average learning outcomes and to address inequality and help disadvantaged learners. Rodriguez-Segura et al. (this issue), analysed interventions in six LMICs and found that non-targeted efforts to improve *foundational* (i.e. early and also essential to other skill) skills have both reduced inequality of learning outcomes and improved average learning outcomes substantially. A similar analysis of a successful foundational literacy program in Rwanda found that the program had the largest impacts on students at the 25th percentile of performance, thus improving average learning outcomes and reducing inequality (Asim, this issue). Better aligning curriculum and instruction with children’s learning levels, so that fewer children fall behind and stop learning, can substantially improve learning outcomes (Kaffenberger and Pritchett, this issue, c).

**Fig. 4:**
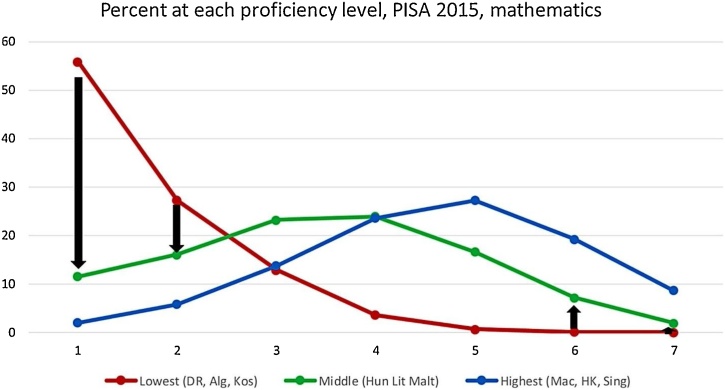
In going from low to middle performance the percentage of children in the two lowest performance levels change the most: dropping from 90 % to just 25 %. *Note*: Percent of children by proficiency level, PISA 2015 mathematics. The red line shows the percentage of children at each level of performance, from lowest (1) to highest (7), for the three countries that have the lowest average performance; the green line shows the same thing for the three countries with middle average performance, and the blue line shows the same thing for the three countries with the highest average performance.

*Source*: Crouch et al. (this issue).

Another paper argues that steepening the learning of the forty percent of students learning the least will go further to improve average learning than focusing on getting high performers to do even better (Crouch et al., this issue). To improve average learning, as many education systems aim to do, systems need to prioritize the “bulging” low performing tail of the distribution (Fig. 4), by ensuring foundational skills for all children.

### 2.3 Girls’ education – including schooling *and* learning – matters for later life outcomes, but a focus on gender will not be sufficient to realize the full benefits of girls’ education

Multiple papers in the Special Issue underscore that the learning crisis is not a gender-specific crisis. Two papers find that the gaps in schooling and learning between girls and boys are small, and sometimes favour girls (Crouch et al., this issue; Kaffenberger and Pritchett, this issue, b). The papers show that achieving gender parity would leave both girls and boys far from education goals.

**Fig. 5:**
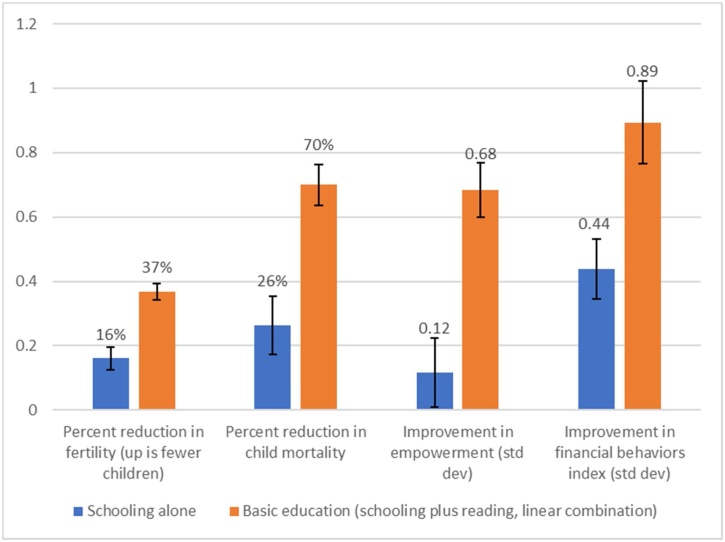
Girls’ basic education, which includes both schooling and literacy, has much larger associations with life outcomes than schooling alone. *Note*: Mean random-effects weighted meta-analysis results from instrumental variables regressions using Demographic and Health Surveys data from 54 countries and 128 survey rounds and Financial Inclusion Insights data from 10 countries. “Schooling alone” is the regression coefficient on years of schooling scaled by 6 to represent primary schooling; “basic education” is the linear combination of the scaled schooling coefficient and a scaled reading coefficient that represent completing primary schooling and going from not being able to read to reading a simple sentence (DHS) or passage (FII) without help.

*Source*: Kaffenberger and Pritchett (this issue, c).

While it is well-established that girl’s *schooling* is associated with positive life outcomes, a paper in this Issue examines the role of *learning* in that relationship across more than 50 countries. It finds that literacy level, in addition to years of schooling, is strongly associated with women’s positive life outcomes including lower child mortality, lower fertility, and greater empowerment (Kaffenberger and Pritchett, this issue, c). While women’s completing primary school alone (without gaining literacy) is associated with a reduction in child mortality of 26 percent, completing primary school *and* gaining basic literacy is associated with a reduction in child mortality of 70 percent, a 44 percentage-point gain over the access-only baseline of 26 percent (Fig. 5). Thus setting the right goals – such as universal foundational skills, rather than equality at low levels – is critical for women’s life outcomes.

### 2.4 Improvements are needed in the early primary years to enable later learning

Because most national and international point-in-time learning assessments occur in late primary or secondary school, much attention goes towards low learning late in children’s schooling. Learning profiles however show that learning often flattens early in the primary school years, as children miss out on foundational skills and cannot engage with more advanced content. In Indonesia, children who have not learned to solve basic mathematics problems by grade six do not gain these skills later in school (Beatty et al., this issue).

Learning profiles for literacy across more than 50 countries show that profiles that start comparatively flat in the early years rarely steepen significantly later (Pritchett and Sandefur, this issue). In Pakistan, primary school students’ learning is often “fragile”, with a sizeable minority of children experiencing learning losses year-to-year (Bau et al., this issue). Addressing low learning requires ensuring adequate mastery of foundational skills early in children’s schooling.

### 2.5 A variety of data sources can be used to analyze, diagnose, and inform approaches to improve learning

There is more data on student learning available now than ever before. A major contribution of this Issue is to show how more can be done with the data that is available.

Multiple papers show how meaningful learning profiles can be analysed from less traditional sources of learning data, which are often more common in LMICs (Kaffenberger, 2019). Some compute learning profiles retrospectively using data from household surveys that include a literacy assessment for adults, such as Pritchett and Sandefur (this issue) with Demographic and Health Survey data, and Kaffenberger and Pritchett (this issue, b) with Financial Inclusion Insights survey data.

Crawfurd (this issue) uses a household survey which assesses children’s learning levels, enabling inclusion of children who have dropped out or otherwise are not in school. Similar contemporaneous cross sections based on household data are used by Akmal and Pritchett (this issue), who use ASER and Uwezo data, and Beatty et al. (this issue), who use a national Indonesian household survey, as well as in the broader literature (Asadullah and Chaudhury, 2014). Johnson and Parrado (this issue) compare ASER learning assessments with a national learning assessment (the National Achievement Survey, or NAS) in India, finding that ASER is more reliable for assessing learning levels and comparisons across states.

In their papers, Rodriguez-Segura et al. (this issue) and Asim (this issue) use programme-related data collected as part of impact evaluations, showing the deeper value that such data can hold beyond judgment of programme success via changes in average learning levels. Crouch et al. (this issue) use more traditional sources of learning data, including from PISA, TIMSS and SACMEQ surveys, but combine analysis across sources to identify common themes. Bau et al. (this issue) use a unique, purpose-driven panel dataset that has been collecting panel learning data on a cohort of children since 2003.

More data would be even more useful, in particular panel data like that analysed by Bau et al. (this issue) that tracks learning in early grades in more countries. And further evidence on how data is used by effective programs to drive management decisions on the ground and inform support to teachers and schools could provide lessons for future programs. But one lesson from the Special Issue is that we can better understand the nature of the learning crisis, and how to act in it, using data that is already available.

## 3 A common understanding of the problem informs “what to do”

Above all, learning profiles can bring those who work in the global education sector to a common understanding of the problem and the priorities needed to address it. Addressing the issue of low learning has been enshrined in global goals since the Jomtien Declaration in 1990. Yet education practice, and behaviors of government officials, teachers, parents, students, donors, and education project implementors, have not changed significantly. Work by the RISE Programme, which many of the papers in this Issue are connected to,^6^ finds that the incentives and accountability structures (including data) in many LMICs education systems remain mostly oriented towards getting children into school.^7^ In coming years the programme will produce tools to support further generation and analysis of learning profiles to inform education system planning for learning oriented policies.

Through showing the scale and nature of low student learning and building nuanced understanding of where low learning is happening and for whom, the papers in this Special Issue show that low learning is not a gender issue, or mostly a rich/poor issue: it is inherently a low-performing-system issue. System level priorities to address low learning, suggested by the findings in this Issue, include clearly prioritizing foundational skills for all children, and prioritizing system-level, learning-oriented policies such as providing learning-goal-oriented support to teachers; developing and deploying curriculum, materials, and instruction aligned with education goals and the learning levels of children; and generating and using learning data to regularly track progress in the early primary years.

The resources needed to achieve these aims may be found by repurposing existing resources in ways that better support learning. For example, redirecting bureaucratic oversight roles to oversee learning performance rather than process compliance, or procuring textbooks that are better aligned with lesson plans and learning objectives (see, for example, Piper et al., 2018). In some contexts, new resources will be needed to ensure adequate resources for learning, and these will need to be used efficiently and effectively (Lewin, 2020).

There are of course other objectives for education (such as social cohesion, parental ability to work, safety, nutritional provision). The message about learning, and addressing the large gaps between national and global aspirations and current achievement, is not to ignore these other goals but to recognise foundational learning as a necessary though not sufficient outcome of schooling.

Before ideas on ‘what works’ to improve student learning can be applied, we must understand the nature and scale of the problem. Learning profiles help to identify a core problem in underperforming education systems. We then need to understand *why* the problem is happening. Only then can we look at ideas that have worked in other contexts and explore how these could, if adapted, work in a new context. As ideas are tested and iterated in new contexts, education actors must keep an eye on the core problem – using learning profiles as a map. Are the learning profiles improving: where, for whom, and how?

## Footnotes

1 From a passage in PIRLS2016, used with permission.

2 The World Bank estimates that post-COVID, nearly two-thirds of children in LMICs (63%) will be in ‘learning poverty’ – i.e. unable to read even the most basic passage by the age of 10, and thus unable to realise the potential of their schooling.

3 This involves adjusting learning profiles to account for higher performing children being more likely to stay in school while lower performing children are more likely to drop out.

4 Indeed in some places things are improving, and there are some case studies of large improvements at scale (Crouch, 2020). But the current levels of learning are so low, and the rate of improvement also so low, that the World Bank (2019) asks a key question and then concludes: “Is [learning poverty] declining rapidly enough to ensure that in 2030, all children will be proficient in reading by age 10, or at least by the end of primary school? The answer is an emphatic ‘No’ if we observe historical rates [of improvement].” Similarly Gustafsson (2020) concludes that, looking at even the best cases under “business as usual”, the empirical “speed limit” for improvement is around 0.08 standard deviations per year with a mean of around 0.04. Given that, as documented by Pritchett (2015), the most needy countries are at least two standard deviations behind, and given that Gustafsson estimates imply an average improvement of 0.4 and best-case scenario of 0.8 standard deviations over the next ten years, it would seem justified to conclude that even where improvements are taking place, if one proceeds with business as usual then, even in the best of cases, the improvement will be nowhere close to enough to close the gap with high-income countries.

5 Note that these papers project limited learning gains, not absolute learning declines from increased schooling. Of the 28 subject-country pairings appearing in both SACMEQ 2000 and 2007, there were only three significant declines in learning achievement no matter how fast the enrolment expansion was during the same period, and in two of those subject-country cases (Mozambique in reading and mathematics), the improvement in the completion rate meant that the production of human capital per child in the population (not per enrolled child), especially for the poorest children, improved significantly. Only Uganda, and only in mathematics, fits the case of “too much access is associated with a drop [not just a low level] in learning” unambiguously. The links between access and learning levels have been explicitly documented only in a few papers – Taylor and Spaull (2015); Crouch and Vinjevold (2006); Le Nestour et al. (2021).

6 We are pleased to see unsolicited papers here too, as indication that a broader network is starting to look at the learning crisis from a systems perspective and to use the methodology of learning profiles.

7 RISE proposes understanding education problems as a series of accountability relationships, summarised in Pritchett (2015), 2015 and illustrated in Atuhurra and Kaffenberger (2020).
